# Support academic access to automated cloud labs to improve reproducibility

**DOI:** 10.1371/journal.pbio.3001919

**Published:** 2023-01-03

**Authors:** Chase Armer, Florent Letronne, Erika DeBenedictis

**Affiliations:** 1 University of Washington, Seattle, Washington, United States of America; 2 Carnegie Mellon University, Pittsburgh, Pennsylvania, United States of America; 3 The Francis Crick Institute, London, United Kingdom

## Abstract

Remotely operated robotic labs, known as cloud labs, have the potential to improve reproducibility in the life sciences. This Perspective investigates the barriers academics face when wanting to use cloud labs and suggests strategies to make this technology more accessible.

## Teaser

Cloud labs, where experiments are executed remotely in robotic facilities, can improve the reproducibility, accessibility, and scalability of experimental biology. Funding and training programs will enable academics to overcome barriers to adopting such technology.

What does the future of experimental biology look like? “Cloud labs” are one ambitious answer to this question. The concept is that one day, rather than working at the bench, scientists will instead program their experiments to be executed at a remote facility using laboratory automation. Cloud labs draw an analogy to cloud computing: Just as cloud computing services give researchers access to open-source libraries of software that can be deployed at scale, on demand, cloud laboratories could enable researchers to execute standardized, robust, scalable, biological protocols in robotically operated laboratories.

Perhaps the most surprising feature of the cloud lab concept is that they already exist. Commercial cloud labs like Strateos and Emerald Cloud Lab have existed for nearly a decade and are primarily used by industry. A few intrepid academics have managed to experiment with commercial cloud labs [1,2], but largely this technology has not had an impact on academic science. This is a shame, as cloud science has the potential to improve the reproducibility, accessibility, and scalability of life science research. With cloud lab tools, academic researchers could more easily reproduce scientific experiments [3], build new methods on top of existing protocols, and share their new open-source contributions to continue the scientific cycle.

Life science experiments are notoriously tricky to make robust and reliable, often flummoxing scientists and slowing progress [4]. In an era characterized by a “reproducibility crisis,” science would benefit from experimenting with new ideas about how to standardize and share methods. Symbolic lab languages [5–7] enable researchers to describe experiments in an intuitive, standardized format that enables biological protocols to be shared in the same manner that open-source software is shared today. Furthermore, cloud labs democratize access to advanced instruments, eliminating the up-front costs associated with purchasing equipment, space, and long-term service contracts. These facilities give academic groups instant access to an extensive variety of specialized instruments that may not be available at their local institution, enabling groups with limited resources to access top-of-the-line equipment.

So, if cloud labs exist and can help to alleviate many of the problems facing life science research today, why do scientists not use them? And what can be done to enable academia to adopt and benefit from the cloud science approach?

**Fig 1:**
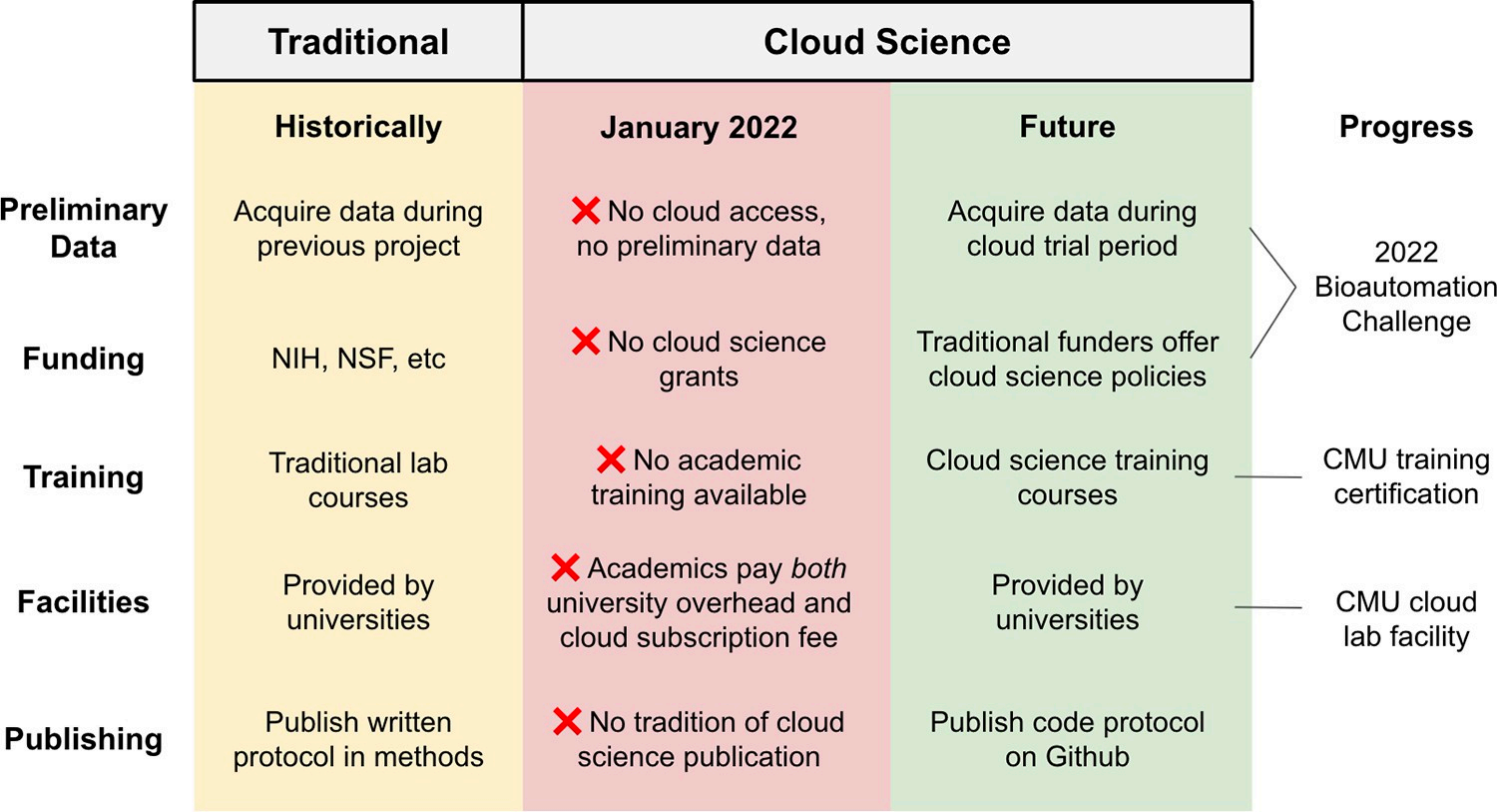
Life cycle of barriers to academic use of cloud labs. Academic scientists face barriers to adopting cloud science at every stage of a normal project’s life cycle. New programs can encourage changes to the ecosystem that make cloud science more accessible to researchers in the long term.

Life science researchers face barriers to adopting cloud technology at every stage of the normal project lifecycle (Fig 1). The first barrier is funding. Current commercial cloud labs have created pricing models that cater to industry customers and are incompatible with the way in which academics usually access facilities and funding. The cost to enter is high (>$250k for general access to Emerald Cloud Lab, or >$100k to automate and run a single method at Strateos), and the contract lengths are long (one year minimum). Cloud lab providers design their pricing to compare favorably for startups who would otherwise need to pay separately for bench space. By contrast, most academic groups already pay overheads to their institution in exchange for facilities access. This effectively double-charges potential academic customers for facilities and contributes to pricing models being unsuitable for academic budgets.

Even if an academic lab were willing to pay such a high price, until recently no grants explicitly allowed use of funds to pay for cloud labs. Traditional funders, such as the NIH or NSF, do not have stated cloud lab policies, and so it is unknown whether these funders would look favorably on cloud lab use in a proposed budget. Furthermore, grants are difficult to acquire without preliminary data, which cannot be acquired when there exists no “trial access” option for accessing cloud laboratories. Without grant support, very few labs possess enough discretionary funds to cover the cost-to-entry for cloud science. As a result, very few labs succeed in overcoming the funding barriers.

New funding mechanisms have started to be designed to sidestep these issues by lowering the cost and risk of cloud lab science for first-time academic users. For example, the 2022 Bioautomation Challenge is a grant program that provides a trial period designed to rapidly expose academic groups to cloud technology. By the end of the program, the academic lab has experience with the platform, an accurate assessment of how much it costs to conduct their particular experiments robotically, and initial data with which they can apply for continued grant funding. The Bioautomation Challenge Partner Program accepts applications from individuals on a rolling basis to receive cloud lab access and training.

Beyond such introductory grant programs, the natural long-term solution would be for universities to provide cloud lab access for their academic labs. Carnegie Mellon University has become the first adopter of this model. Their approach is for the university department to sponsor a cloud lab account, negotiate a bulk rate with a cloud lab provider, and offer access, training, and community support to its members. As academic users become a larger fraction of the cloud lab market, providers will become incentivized to create pricing models that are better designed for academic customers. Primary funders should be encouraged to establish explicit policies encouraging “cloud lab credit” requests in budgets. Until then, these new funding programs are a way for interested scientists to get involved and test the waters of this new paradigm for experimentation.

With more widespread use, we envision a future for life science research in which there is a rich ecosystem of open-source biology protocols and a diverse array of training resources for writing symbolic lab language and executing experiments on the cloud. With these resources, it would be practical and straightforward for new users to incorporate cloud-based science into their research by adapting existing open-source protocols to their specific use case. These researchers could include their newly developed protocols along with their published findings, thereby making this process even more tractable for new users in the future.

Regardless of how productive cloud-based science may one day be, the greatest challenge is in getting started. Currently, there are very few researchers who are experienced with the cloud science paradigm, and few open-source experimental biology protocols that are compatible with cloud laboratories. This forces new users to start from scratch when developing their protocols. Initial efforts like iGEM standard protocols [8] must be greatly expanded to kick-start the open-source software ecosystem. Developing new training programs that teach researchers how to write protocols for cloud-based science will be central to getting the process started. One such training program (developed by Carnegie Mellon University) focuses on three main educational points: understanding the cloud lab (what is a cloud lab, what can and cannot be performed in a cloud lab), how to design and perform scientific experiments in a cloud lab (how to fully or partially translate an experimental workflow from the bench to the cloud lab), and how to combine the power of the cloud lab with other tools such as computational science and open science.

Together, cloud labs represent a new paradigm for interacting with automation and conducting life science experiments. Next-generation automation is no longer reserved for industry but instead is increasingly accessible to academic researchers. This development has the potential to dramatically improve the reproducibility and productivity of life science research. Researchers previously faced barriers to entry at every stage of a project, but new programs are addressing the key challenges of funding and training (Fig 1). While the barriers to academic use of cloud laboratories will require considerable time and effort to overcome, we believe the combination of passionate early adopters with innovative training and funding programs could pave the way for a more reproducible future for life science research.
